# A Systematic Review and Meta-analysis on the Association Between Antiretroviral Therapy Refill Type and Viral Suppression

**DOI:** 10.1007/s10461-026-05136-z

**Published:** 2026-04-17

**Authors:** Oluwaseun Abdulganiyu Badru, Joy Chioma Edeh, Chioma Wisdom Okezie, Oluwafemi Atanda Adeagbo

**Affiliations:** 1 Department of Community and Behavioral Health, College of Public Health, University of Iowa, 145 N. Riverside Dr, Iowa City, IA USA; 2 Ubiquitous Educational Consult Limited, Lagos State, Nigeria; 3 Department of Epidemiology, College of Public Health, University of Iowa, Iowa City, IA USA

**Keywords:** Multi-month dispensing, Differential service delivery, HIV, Viral load, Review

## Abstract

To reduce barriers to viral suppression, people living with HIV (PLWH) are placed on multi-month dispensing (MMD) of antiretroviral therapy medication. However, there are concerns about viral rebound following MMD initiation, and no existing review has compared viral suppression in PLWH on MMD with that in those on monthly refills [standard of care (SoC)]. This meta-analysis compared viral suppression of PLWH on MMD to those on SoC. In February 2025, we searched Embase, PubMed, Scopus, and Web of Science for articles (PROSPERO ID: CRD420251006737). Meta-analysis was performed using RStudio. 1078 articles were retrieved from the databases; 25 met our eligibility criteria (21 met meta-analysis criteria). Compared to PLWH on SoC, viral suppression was better among PLWH on 3MMD or more [risk ratio (RR): 1.17, 1.06–1.30] and 6MMD (RR: 1.24, 1.15–1.34). However, for both 3MMD and 6MMD, the effect sizes were smaller in RCTs than in observational studies. Furthermore, PLWH on 6MMD were less likely to be virally suppressed than those on 3MMD (RR: 0.76, 0.67–0.85). Lastly, compared with SoC, 3MMD or more was significantly associated with viral suppression if defined as < 1000 copies/mL (RR: 1.15, 1.10–1.21), but viral suppression was similar with a stricter cut-off of 50–500 copies/mL (RR: 1.15, 0.93–1.42). Conclusively, PLWH on MMD had lower viral loads than those on SoC, but those on 3MMD were more virally suppressed than those on 6MMD. The advantage MMD has over SoC is insufficient to conclude its superiority. Therefore, MMD implementation should be carefully considered to meet clinic and PLWH’s needs.

## Introduction

HIV is now a manageable chronic disease due to the availability of antiretroviral therapy (ART), and this has increased the number of people living with HIV (PLWH) who lead a healthy and comparable life to the general population [1–3]. To ensure PLWH are retained in care and virally suppressed, they refill their medication monthly at a facility, which is the standard of care (SoC). However, there is evidence of non-suppression globally [4, 5]. More recently, many public health facilities now offer differentiated service delivery models (DSD), defined as service delivery tailored to the unique needs of PLWH to facilitate an uninterrupted HIV care continuum, including home-based ART delivery, refills at private pharmacies, and 3 (3MMD) or 6-month (6MMD) multi-month delivery (MMD), to ensure viral suppression [2, 6, 7].

Multi-month delivery refers to the delivery of ART to clinically stable clients (i.e., those with viral load < 1000 copies per mL of blood) [8]. MMD was recommended in 2016 by the World Health Organization (WHO) for clinically stable or virally suppressed PLWH [9]. However, COVID-19 led to an increase in the adoption of MMD for PLWH, irrespective of viral load status [10]. Post-COVID-19 pandemic healthcare facilities are now only retaining virally suppressed PLWH on MMD, while the non-suppressed engage in the traditional or SoC monthly ART refill [11]. However, there is evidence of viral rebound, defined as viral load detection following previously undetected, among PLWH on MMD [12, 13]. For example, four in 10 PLWH in Tanzania reported viral rebound, and this did not significantly differ between those on MMD and those on monthly ART refill [14]; other studies have reported a viral rebound between 10% and 21% [15, 16]. Therefore, it is crucial to assess whether viral suppression is better among virally suppressed PLWH on MMD than those not on MMD because viral rebound may occur as early as 22 days if ART is interrupted, even among PLWH who are virally suppressed [17].

Previous related DSD reviews have focused on community-based, facility-based, and a mix of both, as well as adherence clubs, on how this may influence HIV treatment outcomes, such as retention in care and viral suppression [18, 19]. However, none of these reviews have compared how MMD and SoC may differ with respect to viral suppression, and they did not compare different MMD types. This review fills this gap.

This review exposure of interest is the type of antiretroviral therapy refill: MMD (i.e., ≥ 3 months) or monthly standard of care (i.e., < 3 months or not on MMD regimen), and the outcome of interest is viral suppression among PLWH [13]. Using analytical observational and experimental studies while excluding previous reviews and non-primary studies, such as letters to editors and commentaries, this meta-analysis aims to assess whether the ART refill schedule (MMD and SoC) is associated with viral suppression. A secondary aim was to assess if the viral load of PLWH of all ages on 3MMD differs significantly from that on 6MMD.

## Methods

We followed the Updated Systematic Review and Meta-Analysis Protocol (PRISMA-P) to conduct this meta-analysis [20] and registered it on PROSPERO (CRD420251006737).

### Eligibility Criteria

We used the population, exposure, and outcome (PEO) to establish eligibility criteria [21]. As such, we included (1) analytical observational and experimental studies that focused on PLWH on either MMD or standard of care and (2) that contain evidence of the association between ART refill type (i.e., MMD and SoC) and viral suppression. We excluded (1) animal studies, (2) previous reviews and non-primary studies, such as letters to the editor and commentaries, (3) primary studies with insufficient data, such as case studies and series, and those that failed to report sufficient ART refill type and viral suppression data. For example, we excluded Hoffman and colleagues’ work because it did not analyze viral load data, since 75% of their participants lacked such data [22].

### Database and Search Strategy

**Fig. 1:**
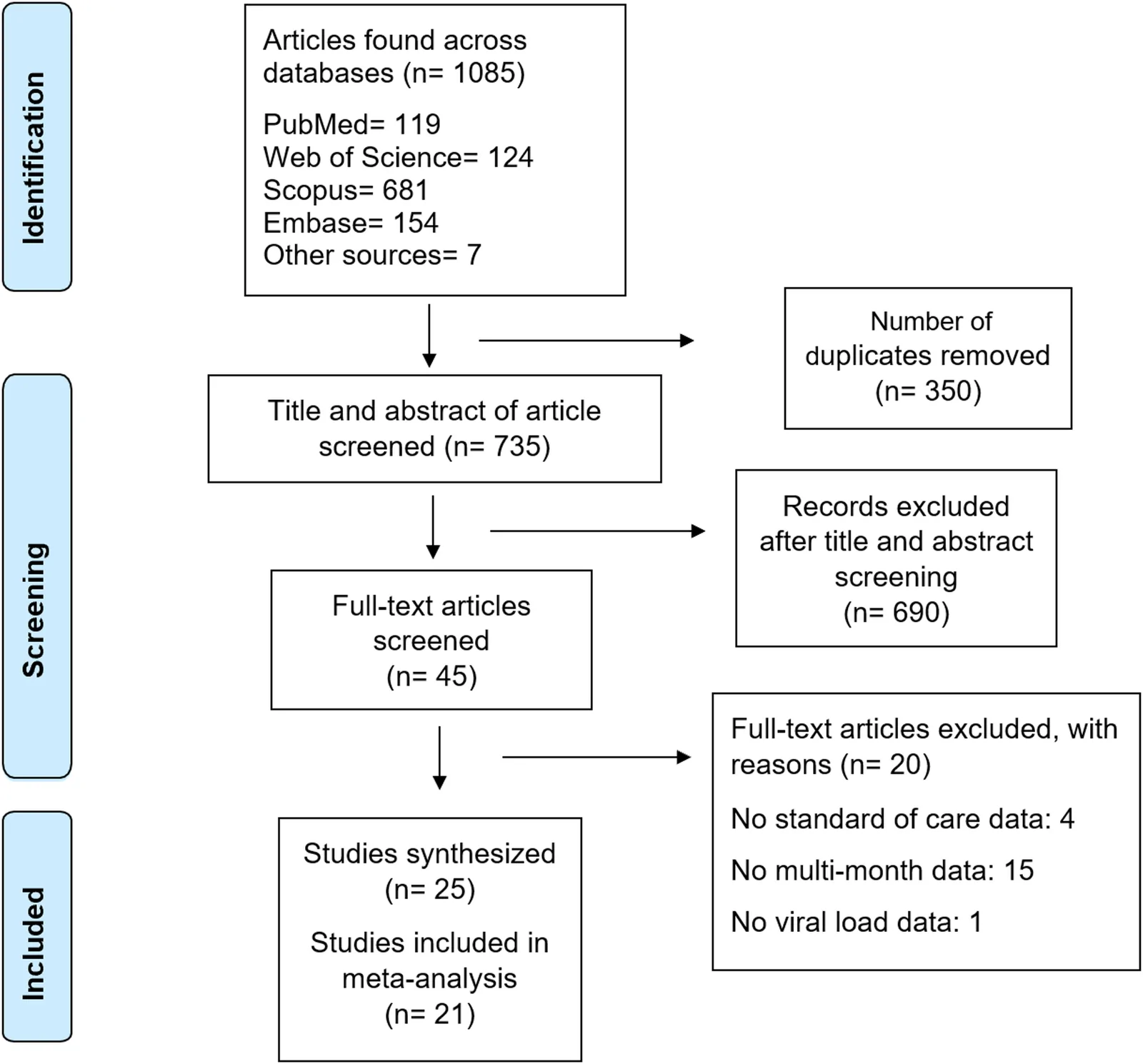
PRISMA flowchart

In February 2025, one reviewer (OAB) systematically searched four databases — Embase, PubMed, Scopus, and Web of Science — from inception, as well as reviewed the first 10 pages of Google Scholar and ResearchGate for additional relevant articles. Additionally, the reviewer searched Google for grey literature. Our search strategy involved identifying keywords related to the PEO, including “people living with HIV,” “multi-month dispensing,” and “viral suppression,” as well as relevant medical subject headings (MeSH) for PubMed and Emtree keywords for the Embase database. In our preliminary search of these databases, we identified keywords and synonymous terms that we added to the Emtree and MeSH. We used parentheses, quotation marks, and truncations where necessary and combined them with Boolean operators. Table 1 lists some of the MeSH terms and keywords used in PubMed (see supplementary material for other database search strategies), and Fig. 1 contains the flowchart of the search strategy.

**Table 1:** Search strategy for PubMed

Keywords	MeSH and Synonyms words
HIV	“HIV“[Mesh] OR “Acquired Immunodeficiency Syndrome“[Mesh] OR “HIV Seropositivity“[Mesh] OR “AIDS virus” OR “HIV positive”
Multi-month dispensing	“multi-month prescription*” OR MMD OR 3MMD OR 6MMD
Viral suppression	“Viral Load“[Mesh] OR “Sustained Virologic Response“[Mesh] OR “viral load” OR “viral suppression” OR “viral load suppression”

We did not limit our search by language. We found only one non-English language article [23] and used Google Translate to translate the text from French to English. Furthermore, to ensure inclusiveness, we attempted to contact authors when we were unable to access their full-text articles or required additional data. Specifically, we contacted the corresponding authors for the full text [24], viral load cut-off [25], and the proportion of virally suppressed PLWH [26]. Harrison et al. [24] responded with their full text, but we did not receive a response from Cavanaugh et al. [26] and Ruhago et al. [25]. Also, we verified the specific MMD type from another group of authors [27]. Additionally, we reviewed the reference lists of the included studies to identify any missed articles during the database search; we did not find any additional articles.

We exported all the articles into Rayyan systematic review manager, and two authors (OAB and JCE) independently reviewed the titles and abstracts to determine their relevance to our research question. Thereafter, both reviewers independently screened the full texts of the studies deemed eligible. We resolved discrepancies through discussion between the two reviewers across all the review stages, and a third reviewer double-checked the abstraction form.

### Data Extraction

One reviewer (OAB), a doctoral candidate with experience in reviews, developed the data abstraction form to facilitate a comprehensive and accurate data extraction process. OAB, JCE, and CWO independently extracted the following information from the included articles: last author’s name and year of publication, study design, country, study setting, population, sampling technique, type of ART refill (i.e., MMD or SoC), viral suppression definition, participants’ characteristics, proportion of PLWH virally suppressed in both groups (i.e., MMD or SoC), and factors associated with viral suppression.

### Quality Assessment

We included cross-sectional, cohort, and randomized controlled trials in our review, and we assessed them using the Newcastle-Ottawa scale (NOS) for cross-sectional studies, the NOS for cohort studies, and the Cochrane Risk of Bias 2.0 tool, respectively. Two reviewers (OAB and JCE) independently appraised the studies with these appraisal tools. We considered cross-sectional and cohort studies with NOS scores ≤ 3, 4–6, and 7–9 to be of low, moderate, and high quality, respectively [28]. For Cochrane RoB 2.0, each domain was rated as low risk of bias, some concerns, or high risk of bias. We considered studies with low risk across all five domains to be high quality, studies with at least one domain rated as having some concerns to be moderate quality, and those with one or more domains rated as high risk of bias to be low quality [29].

### Data Analysis

Before conducting the meta-analysis, we ensured that we did not violate the assumption of independence by selecting only one study when multiple studies used the same dataset. We identified three studies from the same parent intervention [30–32]. One of the studies focused on children only [30], while two others analyzed data of both children and adults [31, 32]. However, in our analysis, we included data from Mugo et al. [31] because it is the most recently published, has the largest sample size, and includes data for both adults and children. In addition, one group of authors had three studies from the same data. One study by Oryokot and colleagues was a preprint [33], which has now been published [34], and another study entirely [35]. We included one of the published studies with sufficient data to compute the risk ratio for meta-analysis [34].

We performed all data analysis using RStudio version 3.6.0+ and the ‘metafor’ and ‘meta’ packages. Statistical analysis included both fixed and random effects models, depending on the observed percentage variability (heterogeneity), as assessed using I² statistics [36]. We considered heterogeneity < 25, 25–49, and > 50% to be low, moderate, and high, respectively [37]. As such, we reported studies with low heterogeneity using fixed-effects models, and the opposite is true for high-heterogeneity observations. However, before conducting the meta-analysis, we converted the effect estimates as needed. Specifically, we converted adjusted odds ratios and risk differences to risk ratios, including confidence intervals when necessary, because most studies reported risk ratios given their cohort and randomized controlled study designs. This conversion ensured study comparability. Notably, we did not switch reference categories because all the studies used SoC as the reference category.

Additionally, we conducted sensitivity analyses to assess the source of heterogeneity and also to assess how important factors may affect the size of the effect estimates [38]. For example, viral suppression, defined as < 1000 copies/mL, may have a different effect estimate than if defined with a stricter cut-off, such as < 200 copies/mL. Specifically, we stratified our analysis by study design, country, comparison of 3MMD and 6MMD with SoC, viral suppression definitions (e.g., <50 and < 1000 copies/mL), as well as publication status (i.e., grey versus published articles), type of study (cross-sectional, cohort, and randomized control trial [RCT]), and population age as defined by the WHO (children only, adults only, and mixed population). We selected these stratifications to investigate potential variability in viral suppression in relation to ART refill type, thereby enabling a robust understanding of how these variables affect viral suppression and a nuanced interpretation of our results. Additionally, to assess publication bias, we visually assessed the funnel plot and double-checked with the Egger’s regression test, where *p* < 0.05 was indicative of publication bias [39]. Finally, we descriptively analyzed the other extracted data, including countries and other study characteristics.

## Results

We found 1,085 articles across the databases and seven from our hand search (total: 1,085). Specifically, we found 154 in Embase, 119 in PubMed, 124 in Web of Science, 681 in Scopus, and seven from other sources (Fig. 1). We removed 350 duplicates and screened the titles and abstracts of the remaining 735 articles. We further excluded 690 articles deemed irrelevant and subjected the remaining 45 to full-text review, resulting in the exclusion of an additional 20 articles. We synthesized the remaining 25 articles; however, we included only 21 in the meta-analysis due to overlapping findings across some studies, which violated the independence assumption (Fig. 1). In all, we analyzed ART refill data for 142,010 PLWH, with 111,130 on MMD and 30,880 on SoC across the 21 studies included in the meta-analysis.

### Study Characteristics

Table 2 indicates that all the studies originated from Africa, except one from Cambodia, Asia [53], with most conducted in Nigeria (*n*=5) [27, 30–32, 41], and Uganda (*n*=4) [33,34, 35, 40]. Three studies from South Africa [42, 44, 48], and two studies from Kenya [13, 50]. One study each from Cameroon [23], Eswatini [46], Lesotho [52], Rwanda [51], Tanzania [25], Zimbabwe [12], Zambia [47]. Lastly, one study combined data from Kenya and Uganda [45], while two other studies were conducted in Lesotho and Zimbabwe [43, 49].

**Table 2:** Characteristics of the included studies

Author and year	Study design	Country	Study setting	Sampling/population	ART refill type and VL definition	VL definition	Participants characteristics	Factors associated for
Ainembabazi et al. 2024 [40]	Cross-sectional	Uganda	118 HIV facilities	Purposive/2864 children and adolescents (i.e., 0–19 years)	MMD: 6 months SoC: ≤1 month	<1000 copies/mL	Sex Female: 1609 (56.2%) Male: 1255 (43.8%) Age (years) Modal age 10–19: 1888 (65.9%)	Crude 1 month (Ref) ≥6MMD: 2.15 (1.48–3.11.48.11) Adjusted 1 month (Ref) ≥6MMD: 2.21 (1.59–3.05.59.05) Adjusted for: age, TB history, duration on ART, ART regimen, and WHO clinical stage
Akpan et al. 2022 [41]	Retrospective cohort study	Nigeria	1 facility	Secondary data/3257 adolescents and adults (age ≥ 15 years)	MMD: 3 months SoC: <3 months	<1000 copies/mL	Sex Female: 2050 (66.6%) Male: 1038 (33.4%) Age Modal group 40+: 1065 (32.7%)	Crude 1 month (Ref) ≥3MMD: 0.72 (0.44–1.20) Adjusted 1 month (Ref) ≥3MMD: 1.02 (0.52–1.82) Adjusted for: age, sex, education level, residency, duration on ART, time to completion of enhanced adherence counseling, facility category, and time to enhanced adherence counseling commencement
Blanco et al. 2024 [13]	Cohort (retrospective)	Kenya	32 government health facilities	2-stage random sampling/3501 adults	MMD: 3 months SoC: <3 months	<1000 copies/mL at month 12	Sex Female: 2415 (69.0%) Male: 1086 (31.0%) Age Median age: 40 (33–48)	Crude <3MMD (Ref) URR: 0.1 (0.0–0.2.0.2) Adjusted <3MMD (Ref) ARR: 0.1 (0.0–0.2.0.2) Adjusted for sex, age, and previous switching between care models
Cassidy et al. 2020 [42]	RCT (unblinded; non-inferiority trial)/Randomization	South Africa	Facility and community	Randomization/2150 adults	MMD: 6 months SoC: 2 months	<400 copies/mL at month 12	Sex Female: 1659 (77.2%) Male: 491 (22.8%) Age Median age: 41.4 (36.5–47.2.5.2)	Risk difference: 5.1% (1.3% to 8.9%)
Ekwuofu et al. 2025 [27]	Cross-sectional	Nigeria	4 facilities	Simple random method/273 adults	MMD: 6 months SoC: <3 months	<1000 copies/mL	Sex Female: 184 (67.4%) Male: 89 (32.6%)	Crude 6MMD (Ref) SoC: 0.662 (0.375–1.168) Adjusted 6MMD (Ref) SoC: 0.211 (0.098–0.455) Adjusted for: gender, marital status, place of residence, and adherence
Fatti et al. 2020 [12]	RCT (three-arm, unblinded, parallel, non-inferiority, cluster)	Zimbabwe	Facility and community	Randomization/4800 adults	MMD: 6 months (community) SoC: 3 months (facility) 3 months (community)	<1000 copies/mL at month 12	Sex Female: 3463 (72.2%) Male: 1332 (27.8%)	Adjusted 3MMD (Facility; Ref) 3MMD (community) RR: 1.4 (0.2–10.9.2.9) 6MMD (community) RR: 0.1 (0.02–0.60.02.60) Adjusted for randomization stratum
Fatti et al. 2021 [43]	RCT (three-arm, unblinded, parallel, non-inferiority, cluster)	Zimbabwe and Lesotho	Facility and community	Randomization/599 adults	MMD: 6 months (community) SoC: 3 months (facility) 3 months (community)	<1000 copies/mL at month 12	Sex Female: 381 (63.6%) Male: 218 (36.4%) Age Median age (IQR): 39.8 (32.8–49.6)	Crude 3MMD (Facility; Ref) 3MMD (community) RR: 0.99 (0.97–1.02.97.02) 6MMD (community) RR: 0.99 (0.96–1.01.96.01) Adjusted 3MMD (Facility; Ref) 3MMD (community) RR: 0.98 (0.92–1.03.92.03) 6MMD (community) RR: 0.98 (0.95–1.00.95.00) Adjusted for primary healthcare clinic/hospital-based facility and rural/urban geolocation
Fox et al. 2019 [44]	RCT (unblinded; cluster)	South Africa	Facility and community	Randomization/569 adults	MMD: 6 months SoC: 2 months	<400 copies/mL at month 12	Sex Female: 410 (72.0%) Male: 159 (28.0%) Age Modal group 30–39: 208 (37.0%)	Crude DiD: −0.5% (−7.5%−6.6%) Adjusted DiD: −1.0% (−12.2%−10.1%) Adjusted for clustering by site
Hickey et al. 2020 [45]	RCT (cluster)	Kenya and Uganda	32 communities	Randomization/6190 adolescents and adults (age ≥ 15 years)	MMD: 3 months SoC: 1 month	< 500 copies/mL at year 3	Sex Female: 4112 (66.4%) Male: 2078 (33.6%)	Crude RR: 1.03 (1.01–1.06.01.06)
Hlophe et al. 2025 [46]	Cross-sectional	Eswatini	Facility	National record 911 adolescents (age 10–19 years)	MMD: 3 and 6 months SoC: 1 month	< 1000 copies/mL	Sex Female: 551 (60.5%) Male: 360 (39.5%) Age Modal age 15–19: 606 (66.5%)	Crude Monthly (Ref) OR: 0.22 (0.05–0.92) Adjusted Monthly (Ref) AOR: 1.23 (0.67–2.23) Adjusted for age, sex, region, facility ownership, ART duration, ART regimen, and WHO staging
Lamba et al. 2025 [47]	Cross-sectional	Zambia	1 facility	Secondary data/10,758 adolescents and adults (age ≥ 15 years)	MMD: 3–5, ≥6 months SoC: <3 months	< 1000 copies/mL	Sex Female: 7121 (62.2%) Male: 3637 (33.8%) Age Modal age: 50+ 2019 (18.8%)	Crude <3 months (Ref) OR for 3-5MMD: 19.48 (14.73, 25.76) OR for 6MMD: 8.60 (6.46, 11.45) Adjusted <3 months (Ref) OR for 3-5MMD: 18.26 (13.47, 24.74) OR for 6MMD: 22.14 (15.45, 31.72) Adjusted for sex, age, ART regimen, and years on ART
Lewis et al. 2023 [48]	Cohort (retrospective)	South Africa	59 public facilities	27,148 adults	MMD: 12 months MMD: 6 months	< 50 copies/mL at month 12	Sex Female: 18,906 (69.6%) Male: 8242 (30.4%) Age Median age (IQR): 39 (33−46)	Crude 6MMD (Ref) RR: 1.01 (1.00–1.02.00.02) Adjusted 6MMD (Ref) ARR: 1.00 (0.99–1.01.99.01) Adjusted for sex, age, years on ART, ART regimen, previous community ART use, and month
Lopes et al. 2021 [49]	RCT (cluster, *n*=2)	Zimbabwe and Lesotho	30 facilities	Randomization/10,136 adults	MMD: 6 months MMD: 3 months	< 1000 copies/mL at month 12	Sex Females: 7002 (69.1%) Male: 3134 (30.9%)	Adjusted 3MMD facility (Ref) 3MMD community: 1.0 (1.0-1.0.0.0) 6MMD community: 1.0 (1.0-1.0.0.0) Adjusted for place of residence, hospital/primary healthcare facility, district, and country
Kengni et al. 2024 [23]	Cohort (retrospective)	Cameroon (French)	1 health facility	519 adolescents and adults (age ≥ 15 years)	MMD: 3 months SoC: 1 month	< 1000 copies/mL at first VL test (time not stated)	Sex Male-to-female ratio: 0.53 Age Modal group 30–45: 223 (43.0%)	Crude Monthly (Ref) OR: 2.55 (1.53–4.25.53.25) Adjusted Monthly (Ref) ARR: 3.06 (1.74–5.37.74.37) Adjusted for alcohol use, tobacco use, and treatment stoppage
Mugo et al. 2023 [30]	Cohort (retrospective)	Nigeria	73 facilities (in 11 states)	1584 children (0–15 years)	MMD: ≥3 months SoC: <3 months	<1000 copies/mL closest to months 12	Sex Female: 771 (49.0%) Male: 813 (51.0%) Age Modal age ≥ 10: 734 (48%) Range: 0–15	Crude Soc (Ref) RR: 1.14 (1.07–1.23.07.23) Adjusted Soc (Ref) ARR: 1.05 (0.94–1.18.94.18) Adjusted for duration on ART, sex, baseline VL, and duration of follow-up
Mugo et al. 2024 [31]	Cohort (retrospective)	Nigeria	63 facilities	84,776 children and adults (age ≥ 10 years)	MMD: 3–5, ≥6 months SoC: <3 months	<1000 copies/mL from 6 months	Sex Female: 57,044 (67.0%) Male: 27,732 (33.0%) Age Modal age 30–39: 30,250 (36.0%)	Adjusted Soc (Ref) ARR for 3-5MMD: 2.8 (2.30–3.47) ARR for 6MMD: 6.3 (5.11–7.69) Adjusted for sex, age, regions, duration on ART, and facility type
Oryokot et al. 2024 [33]	Cross-sectional (Mixed methods; quantitative was secondary abstraction from records)	Uganda	2 facilities	Census sampling/533 children and adolescents (10–17 years)	MMD: 3–5, ≥6 months SoC: <3 months	VL: <200 copies/mL within 12 months	Sex Female: 289 (54.2%) Male: 244 (45.8%) Age Modal age 15–18: 334 (62.7%)	Crude Soc (Ref) OR for 3-5MMD: 1.977 (1.105–3.538) OR for 6MMD: 3.487 (1.852–6.566) Adjusted Soc (Ref) AOR for 3-5MMD: 1.666 (0.392–7.084) AOR for 6MMD: 3.095 (0.615–15.585) Adjusted for age, adherence score, weight, duration on ART, education, relationship with caregiver, caregiver VL, ART line, distance to facility, orphaned and vulnerable children, and differentiated service delivery model
Oryokot et al. 2025 [34]	Cross-sectional (Mixed-methods; quantitative was secondary abstraction from records)	Uganda	2 facilities	Census sampling/533 children and adolescents (10–17 years)	MMD: 3–5, ≥6 months SoC: <3 months	<1000 copies/mL within 12 months	Sex Female: 289 (54.2%) Male: 244 (45.8%) Age Modal age 15–18: 334 (62.7%)	Crude Soc (Ref) OR for 3-5MMD: 3.549 (1.859–6.774) OR for 6MMD: 7.494 (3.551–15.818) Adjusted Soc (Ref) AOR for 3-5MMD: 3.403 (1.449–7.991) AOR for 6MMD: 5.553 (2.078–14.842) Adjusted for adherence score, weight, current ART line, and Differentiated service delivery model
Oryokot et al. 2025 [35]	Pre- and post-intervention	Uganda	2 facilities	Census sampling/1043 children and adolescents (10–17 years)	MMD: 3–5, ≥6 months SoC: <3 months	<1000 copies/mL within 12 months	Sex Female: 560 (53.7%) Male: 483 (46.3%)	Adjusted Soc (Ref) AOR for 3-5MMD: 5.46 (1.66–17.93) AOR for 6MMD: 11.65 (2.93–46.34) Adjusted for sex, study centers, adherence score, current differentiated service delivery model, benefit from orphaned and vulnerable children services, and intervention period
Oyuga et al. 2024 [50]	Cohort (retrospective)	Kenya	43 facilities	963 children (2–9 years)	MMD: 3 months SoC: <3 months	<1000 copies/mL at least 3 months after the first visit	Sex Female: 488 (50.7%) Male: 475 (49.3%) Age Modal age 5–9: 628 (65.2%)	Crude Soc (Ref) OR: 1.25 (1.15–1.38.15.38) Adjusted Soc (Ref) AOR: 1.22 (1.12–1.34.12.34) Adjusted for age, sex, time on ART, ART regimen, and WHO clinical stage
Ross et al. 2024 [51]	RCT (unblinded)	Rwanda	3 facilities	Randomization/90 adolescents and adults (age ≥ 15 years)	MMD: 3 months (group 1: 1 VL; group 2: 2 VL) SoC: 1 month	<200 copies/mL at month 12	Sex Females: 60 (66.7%) Male: 30 (33.3%) Age Mean age (SD): 30.8 (10.1)	Adjusted Soc (Ref) AOR group 1: 1.12 (0.90–1.41) AOR group 2: 1.11 (0.88–1.38) Adjusted for educational attainment and internalized stigma
Ruhago et al. 2022 [25]	Cross-sectional	Tanzania	44 facilities	Multistage (Stratified random sampling and simple random sampling)/83,142 adolescents and adults (age 15–49)	MMD: ≥3 months SoC: <3 months	--	Sex Female: 54,404 (65.4%) Male: 28,738 (34.6%) Age Modal age ≥ 5: 99.2%	
Semo et al. 2023 [32]	Cohort (retrospective)	Nigeria	75 facilities	19,859 children and adults (≥ 10 years)	MMD: ≥3 months SoC: <3 months	<1000 copies/mL at month 6–12	Sex Female: 12,664 (64.0%) Male: 7195 (36.0%) Age Modal age > 24: 15,742 (79.0%)	VL (6 months) Adjusted Soc (Ref) ARR: 1.01 (0.97–1.04) VL (12 months) Adjusted Soc (Ref) ARR: 1.02 (0.98–1.05) Adjusted for age, gender, ART regimen, and the period of enrollment, and facility clustering
Tukei et al. 2020 [52]	RCT (three-arm, non-inferiority, cluster)	Lesotho	Facility and community	Randomization/5336 adults	MMD: 6 months (community) SoC: 3 months (facility) 3 months (community)	<1000 copies/mL at month 12	Sex Female: 3539 (66.3%) Male: 1797 (33.7%)	Crude 3MMD (Facility; Ref) 3MMD (community) RR: 0.70 (0.35–1.41.35.41) 6MMD (community) RR: 0.84 (0.36–1.93.36.93) Adjusted 3MMD (Facility; Ref) 3MMD (community) RR: 1.15 (0.54–2.47.54.47) 6MMD (community) RR: 1.30 (0.57–2.93.57.93) Adjusted for district and randomization stratum
Yi et al. 2025 [53]	Quasi-experimental study	Cambodia	20 facilities and communities (10 per arm)	3067 adolescents and adults (age ≥ 15 years)	MMD: 3–6 months SoC: 1 month	<1000 copies/mL at month 18	Sex Female: 1830 (59.7%) Male: 1151 (40.3%)	

MMD: multi-month dispensing; SoC: standard of care; VL: viral load; ARR: CI: confidence interval; SD: Standard deviation; ART: antiretroviral therapy; IQR: interquartile range; RCT: randomized control trial; SRS: Simple random sampling

Most of the studies were experimental (*n*=10) with eight randomized controlled [12, 42–45, 49, 51, 52], one quasi-experiment [53], and pre- and post-intervention study [35]. Eight studies were cohort designs [13, 23, 30–32, 41, 48, 50], and seven were cross-sectional [25, 27, 33, 34, 40, 46, 47].

Eighteen of the 25 studies obtained data from PLWH in one or more health facilities [13, 23, 25, 27, 30–33, 35, 40, 41, 46–51]. Six studies focused on PLWH in health facilities and communities [12, 42–44, 52, 53], and a Kenyan-based study focused on PLWH in the community, although they linked participants to clinics [45].

Regarding how the studies defined MMD, six studies defined it as 3 MMD of ART dispensing [13, 23, 41, 45, 50, 51], nine as 3 MMD or more [25, 30–35, 47, 53], and eight studies as 6 MMD of ART dispensing [12, 27, 40, 42–44, 49, 52]. One study each was 3 and 6 MMD [46], and one was 12 MMD [48]. Furthermore, 18 of the 25 studies defined viral suppression as viral load < 1000 copies/mL [12, 13, 23, 27, 30–32, 34, 35, 40, 41, 43, 46, 47, 49, 50, 52, 53], one as < 500 copies/mL [45], and two each as < 400 copies/mL [42, 44] and two as < 200 copies/mL [33, 51], one as < 50 copies/mL [48], and one study was not explicit [25].

### Association Between ART Refill Type and Viral Suppression

#### 3MMD or more Versus SoC

**Fig. 2:**
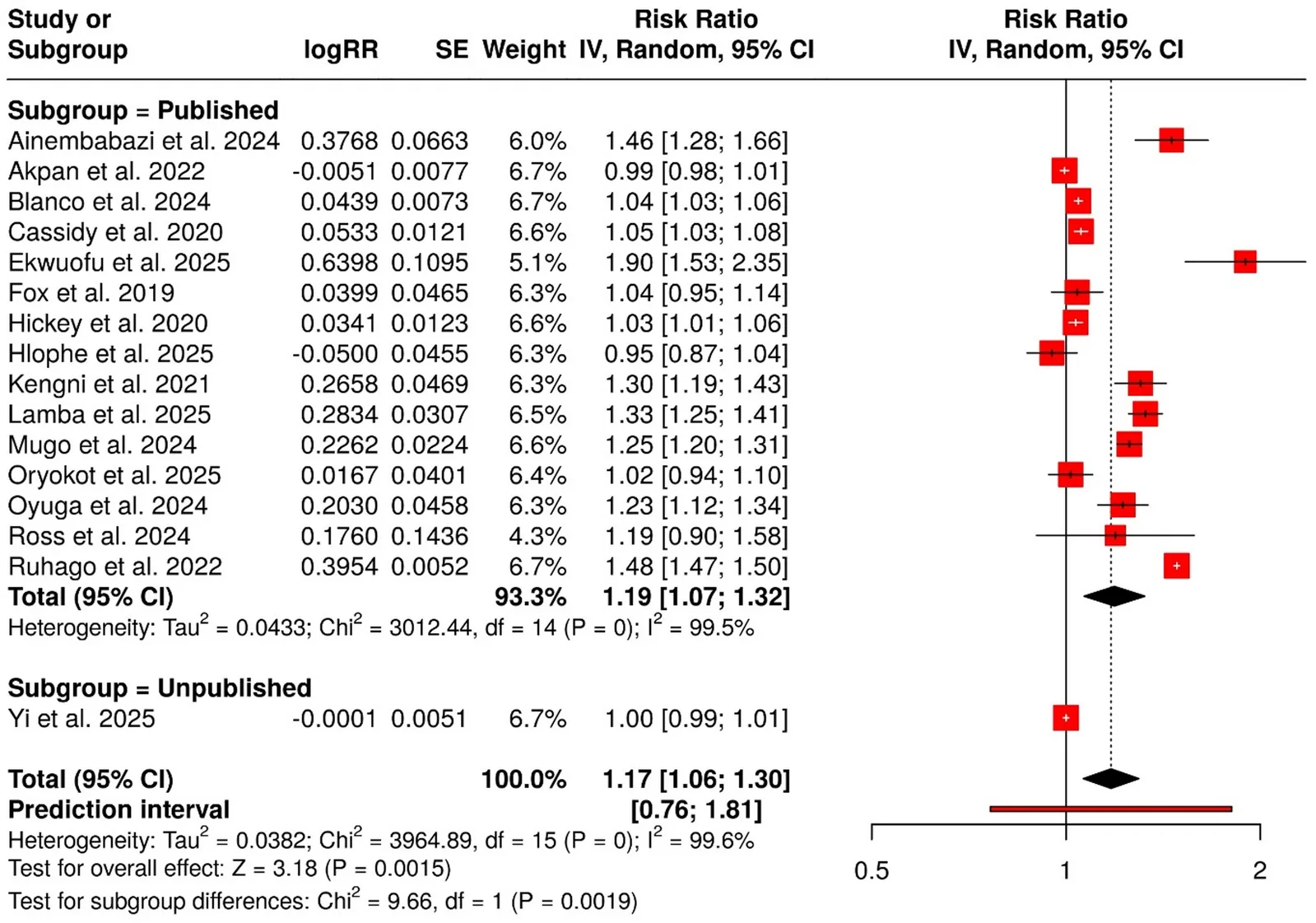
Forest plot showing the association between ART refill type and viral suppression, and by publication status

**Fig. 3:**
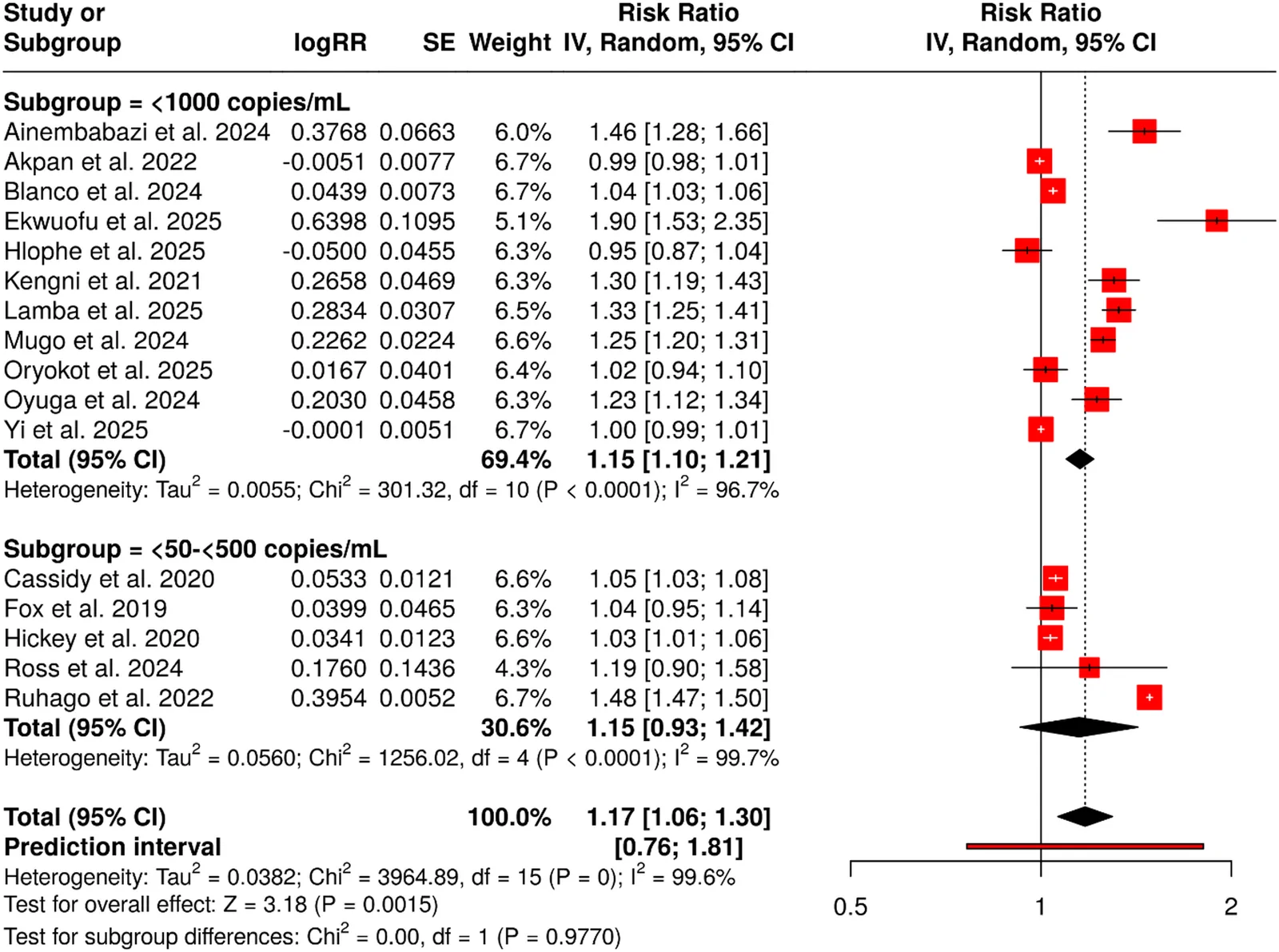
Forest plot showing the association between ART refill type and viral suppression by viral suppression cut-off

Figure 2 shows that PLWH on MMD were significantly 17% more likely to be virally suppressed, although with a high heterogeneity (risk ratio [RR]: 1.17, 1.06–1.30.06.30; I^2^: 99.6%). We isolated the only unpublished literature to see whether the estimates would change; the result remains unchanged (see Fig. 2). However, our subgroup analysis revealed nuances. First, studies that defined viral suppression as < 1000 copies/mL showed that the MMD group was 15% more likely to achieve viral suppression than the SoC group (RR: 1.15, 1.10–1.21.10.21; I^2^: 96.7%). However, viral suppression was similar between the MMD and SoC groups when the viral load cut-off was defined as between < 50 and 500 copies/mL (RR: 1.15, 0.93–1.42.93.42; I^2^: 99.7%; Fig. 3).

**Fig. 4:**
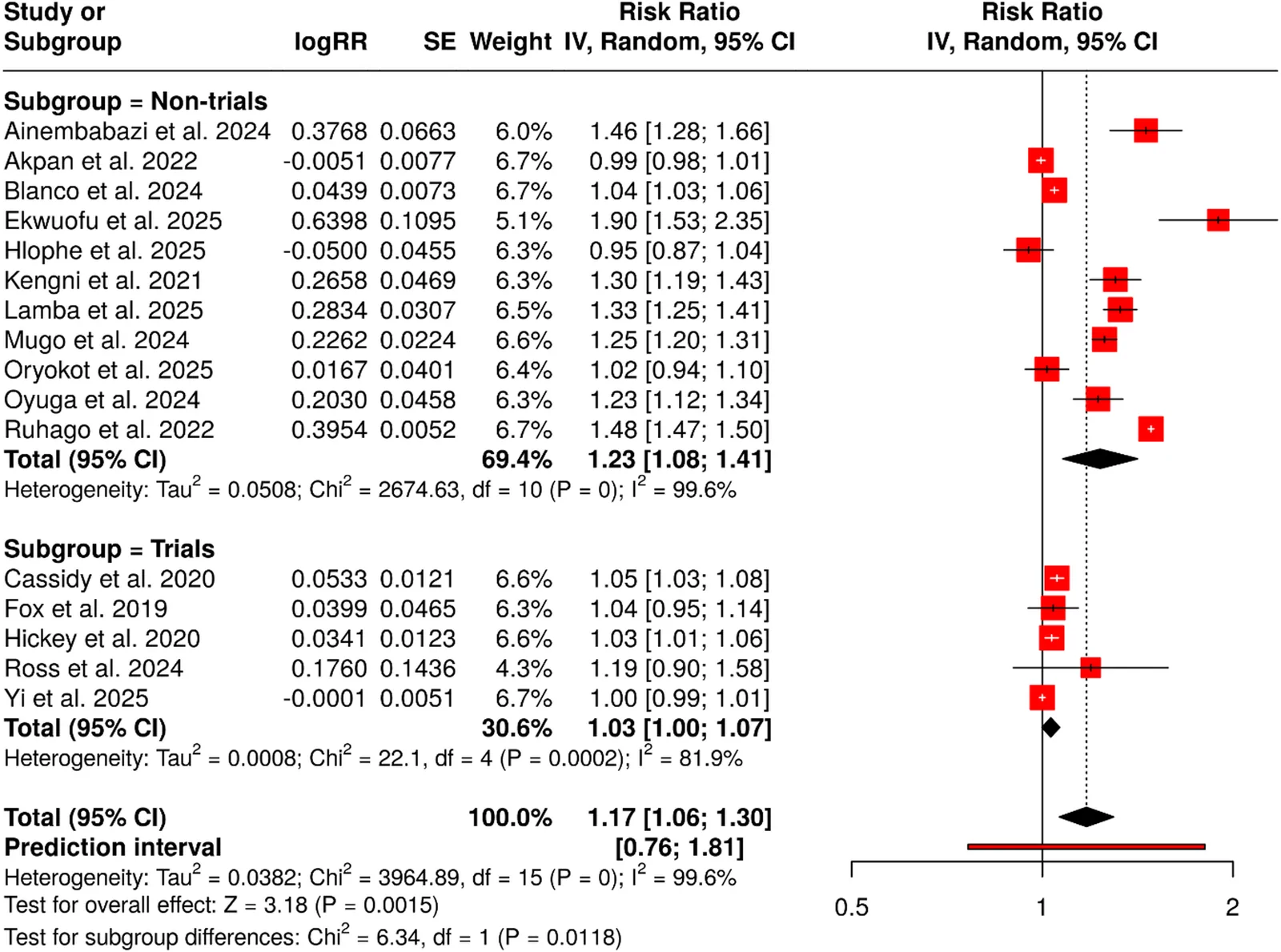
Forest plot showing the association between ART refill type and viral suppression by study design

Second, Fig. 4 shows that the pooled effect size of viral suppression favoring the MMD group decreases as the study design strength increases. Cross-sectional studies showed the highest effect size of 1.30 (1.10–1.54.10.54), followed by cohort studies at 1.15 (1.06–1.23.06.23), and RCTs at 1.05 (1.03–1.06.03.06). Furthermore, we collapsed the studies into non-trials (i.e., cross-sectional and cohort) and trial studies (i.e., quasi experiments and RCTs) in Table 3 and found a similar pattern with observational studies having a higher effect size (RR: 1.23, 1.08–1.41.08.41; I^2^: 99.6%) than trials (RR: 1.03, 1.00–1.07.00.07; I^2^: 81.9%). Also, MMD in the facility alone yielded a higher viral suppression in the MMD group than the SoC (RR: 1.21, 1.08–1.36.08.36; I^2^: 99.7%), but the effect size reduced when PLWH had the flexibility of refilling ART in the facility or community (RR: 1.04, 1.03–1.06.03.06; I^2^: 0%). Lastly, the odds of viral suppression were higher for the MMD group when assessed at 12 months or later (RR: 1.03, 1.01–1.06.01.06; I2: 83.5%), but not at 12 months (RR: 1.14, 0.97–1.34.97.34; I^2^: 93.9%). Adults on MMD were significantly more likely to be virally suppressed than those on SoC (RR: 1.09, 1.03–1.16.03.16; I^2^: 89.9%); we observed the same with children/adolescents, but this was not significant (RR: 1.14, 0.97–1.35.97.35; I^2^: 92.0%). However, our analysis for mixed populations (i.e., children/adults and adolescents/adults) reported better viral suppression for the MMD group (RR: 1.19, 1.01–1.39.01.39; I^2^: 99.8%).

**Table 3:** Subgroup analysis for multi-month type, study design, population, and viral suppression definitions

Keywords	MeSH and synonyms words
HIV	“HIV“[Mesh] OR “Acquired Immunodeficiency Syndrome“[Mesh] OR “HIV Seropositivity“[Mesh] OR “AIDS virus” OR “HIV positive”
Multi-month dispensing	“multi-month prescription*” OR MMD OR 3MMD OR 6MMD
Viral suppression	“Viral Load“[Mesh] OR “Sustained Virologic Response“[Mesh] OR “viral load” OR “viral suppression” OR “viral load suppression”

*Bolded risk ratios are significant; SoC: standard of care; RCT: randomized control trial; #: adolescents/adults or children/adults

#### 6MMD Versus SoC

**Fig. 5:**
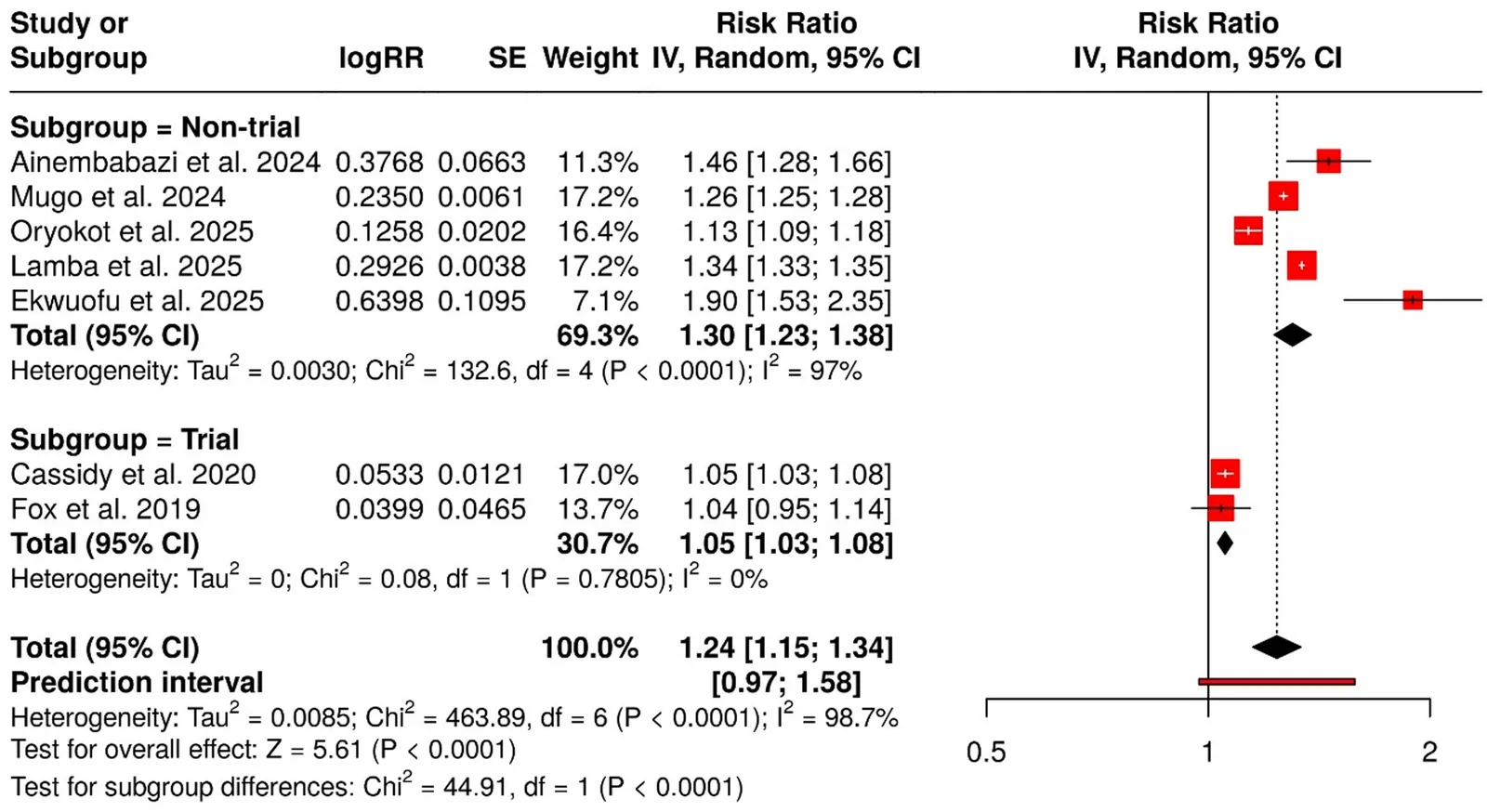
Forest plot showing 6 MMD vs. SoC and association with viral suppression by study design

Figure 5 shows 6MMD and SoC with respect to viral load suppression. PLWH on 6MMD were 24% more likely to be virally suppressed (RR: 1.24, 1.15–1.34.15.34; I^2^: 98.7%) than those on SoC. Furthermore, similar to 3MMD or more, non-trials had a higher effect size than trials.

#### 3MMD Versus 6MMD and 6MMD vs. 12MMD

**Fig. 6:**
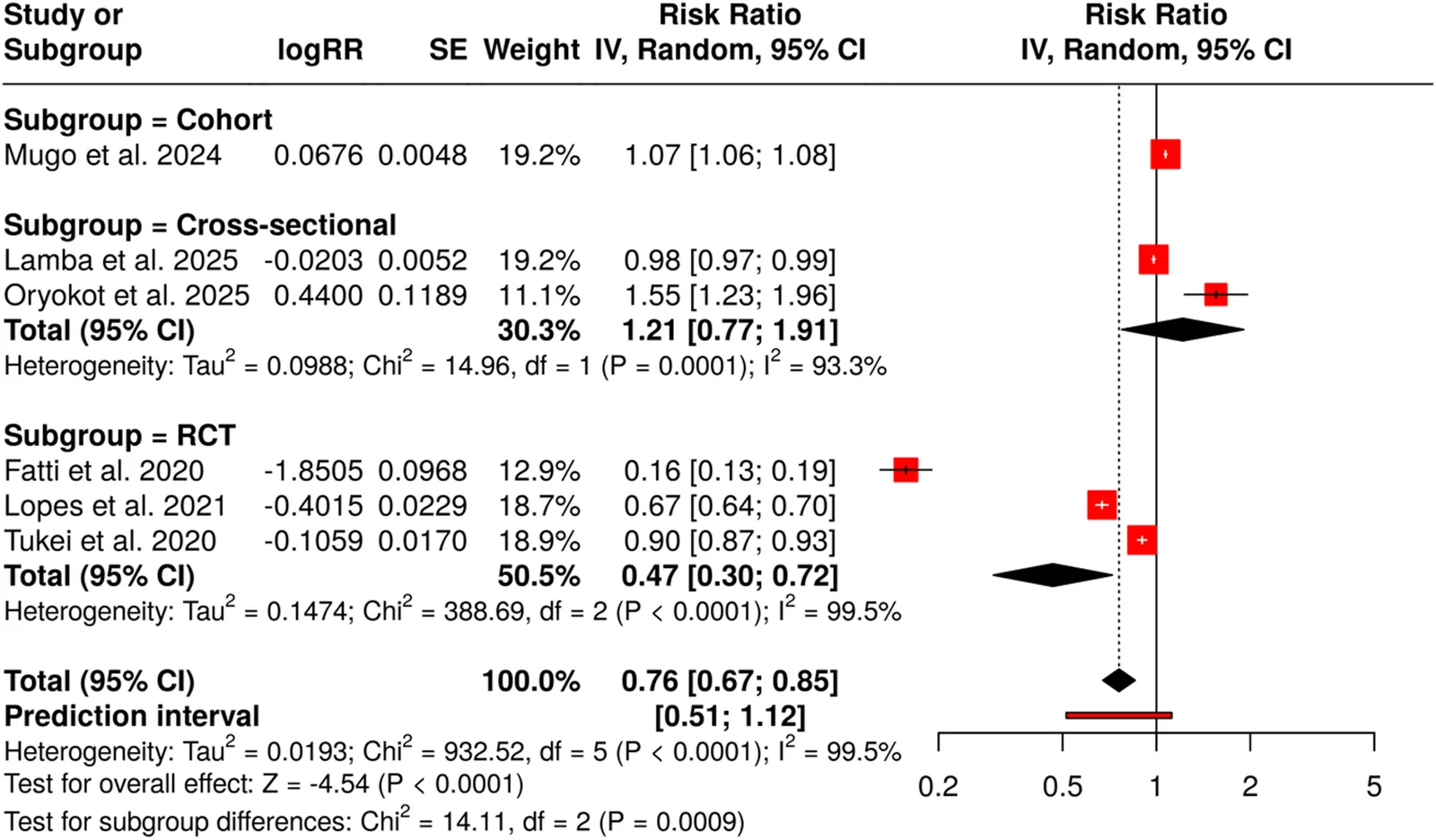
Forest plot showing 3 MMD vs. 6 MMD and association with viral suppression by study design

In Fig. 6, we compared the viral suppression rates of PLWH on 3MMD with those on 6MMD, and, interestingly, cross-sectional (RR: 1.21, 0.77–1.91.77.91) and cohort (RR: 1.07, 1.06–1.08.06.08) studies favored the 6MMD group for viral suppression over the 3MMD group. However, RCTs showed the reverse, with the 6MMD group less likely to be virally suppressed (RR: 0.47, 0.30–0.72.30.72; I^2^: 99.5%), and the effect of RCTs was sustained in the overall pooled effect of all the studies, such that those on 3MMD were 24% more likely to be better suppressed (RR: 0.76, 0.67–0.85.67.85; I^2^: 99.5%). One study that compared 6MMD to 12MMD found that PLWH on either regimen had similar viral loads after adjusting for confounders (RR: 1.00, 0.99–1.01.99.01) [48].

#### Critical Appraisal and Risk of Bias

We judged all the studies to be of high quality. However, we noticed that the two cross-sectional studies did not report the response rate [33, 40]. Additionally, one of the studies [33] did not justify the reason for their sample size, which contributed to the deduction of points. Furthermore, among the cohort studies, we observed that follow-up adequacy [23] and unclear information on whether the outcome was not present at the start of the study [30, 50] resulted in some points being deducted. Regarding the risk of bias, the funnel plot suggests no publication bias, and Egger’s regression test supports this (t = 2.16; p-value = 0.06).

## Discussion

In this meta-analysis, we assessed whether viral suppression would be better among PLWH on MMD than among those on SoC, and whether being on 3MMD suppresses viral load more than being on 6MMD. To our knowledge, this is likely to be one of the first meta-analyses to evaluate the association between viral suppression and MMD. All 25 studies, except one, originated from Africa, indicating a need for more studies from outside the region.

We judged all the studies to be high quality, even though not all had the highest possible appraisal score on the assessment tools. We identified issues with sample size justification [33] that future cross-sectional studies may want to address, as well as unclear details regarding whether the outcome was not present at the commencement of the study [29, 48], which should be clarified in future studies. Although the funnel plot and Egger’s regression test did not indicate evidence of publication bias, inherent biases exist in different study designs. For example, cross-sectional studies are susceptible to recall bias, while cohort studies and trials are prone to loss to follow-up, which may have distorted our results. However, the lack of publication bias in our study may be partly explained by the fact that the assessment of MMD is less likely to be unbiased because all the studies operationalized it as three or more bottles of ART during pick up, and viral suppression assessed with blood work; however, we cannot rule out systematic measurement bias and errors related to when they assessed viral load (i.e., timing) and how these studies defined viral suppression. Variability in how timing was measured and in how studies defined viral suppression may have biased our findings.

Our study shows that PLWH on MMD (both 3MMD and 6MMD) were significantly more likely to be virally suppressed than those on SoC, suggesting that MMD may be superior to SoC for achieving viral suppression. This finding supports our study aim of a significant association between ART refill type and viral suppression. However, it is noteworthy that our study design subgroup analysis revealed that, despite better viral suppression in the MMD group compared with SoC, the effect size in experimental studies was smaller than in observational studies (RR of 1.23 vs. 1.03). This difference may be due to residual confounding in the observational studies. Usually, health providers place PLWH (e.g., excessive alcohol users) with a perceived higher risk of non-adherence and treatment failure on monthly dispensing (i.e., SoC) for closer monitoring and more clinically stable PLWH on MMD [54, 55]. These confounding factors may lead to an overestimation of viral suppression in observational studies, whereas in experimental studies, where PLWH are randomly assigned to refill groups, such cofounders are eliminated, with a smaller event size more likely, as shown in our study.

We recommend that health facilities return to the WHO criteria for placing PLWH on MMD (i.e., only those who are virally suppressed). Although we recognize that this guideline may not be feasible in settings overwhelmed by workload due to staff shortages or the distance PLWH need to travel to reach the facility [56], it is imperative to ensure HIV clinics follow the guidelines to assess MMD’s impact on viral suppression, which will also allow for a comparative analysis of possible issues with other settings. Also, HIV clinics must consider factors that may affect their successful implementation of MMD, such as the cost of procuring medications or securing donations. While storing medications for MMD may reduce patient load in pharmacies, HIV clinics should consider how more medication inventory may burden them, particularly with storage. In essence, clinics that intend to commence MMD should weigh the pros and cons for themselves and their clients.

Interestingly, we observed that PLWH on 6MMD were less likely to be virally suppressed than those on 3MMD, supporting our secondary study aim. In other words, placing PLWH on 3MMD yields better viral suppression than on 6MMD. More interestingly, observational studies (cross-sectional and cohort) tend to favor 6MMD over 3MMD, but the direction changes in RCTs, and the RCTs’ effect ensures that the overall pooled effect size favors 3MMD over 6MMD despite all studies having somewhat equal weight in the analysis (see Fig. 6). This perhaps shows the importance of RCTs. However, it is important to reflect on why 3MMD achieves better viral suppression than 6MMD. There are plausible explanations for this finding. First, a three-monthly engagement allows for more frequent contact with the health professionals who can reinforce adherence counseling, especially those PLWH who are newly stable on ART [57]. Second, PLWH on 3MMD are less likely to run out of ART medication compared to those on 6MMD, which reduces the risk of medication mismanagement, such as loaning medication to family or friends living with HIV [58]. Even if they run out of ART medication, they can access medication more quickly due to a shorter timeframe.

Moreover, how studies defined viral suppression influenced whether PLWH were considered suppressed. Specifically, we found that when viral suppression was defined as < 1000 copies/mL, those on MMD had a 15% likelihood of viral suppression compared to those on SoC, but viral suppression was similar between those on 3MMD or more and those on SoC with a stricter viral suppression cut-off (< 50–500 copies/mL). The use of varying definitions of viral suppression reflects how countries or even facilities choose to classify it, but these findings indicate that the effect of MMD on viral suppression depends on the definition used. It therefore implies that with a stricter viral suppression goal, MMD offers no advantage over a monthly refill. This should be a concern because it indicates that MMD requires closer monitoring if the facility defines viral suppression as < 1000 copies/mL.

This study is not without limitations. First, almost all the studies in this review originated in Africa, which limits our findings to this region. Second, we primarily utilized the adjusted effect sizes (odds and risk ratios) from individual studies, converting odds to risk ratios as necessary. We did not have control over the variables the studies adjusted for, which may have influenced their effect sizes and our findings. Lastly, our SoC definition was a monthly ART refill. However, some studies defined SoC as allowing up to two monthly refills, which may have influenced our findings, as technically, two monthly refills are also MMD. However, we adhered to the WHO recommendation of providing three or more ART refill bottles [59].

## Conclusions

Our findings indicate that PLWH on MMD are more likely to be virally suppressed than PLWH on SoC; this is even more pronounced when PLWH receive ART refills only in the health facility, compared with when their health facility allows facility- and community-based refills. However, the advantage MMD has over SoC in terms of viral suppression was lost when a stricter cut-off of < 50 to < 500 copies/mL was used to define viral suppression. Also, the larger effect size of MMD over SoC in observational studies compared to RCTs may be confounded by the lack of randomization of PLWH to treatment, which may overstate MMD’s dominance over SoC. As such, the current evidence should be interpreted with caution. Furthermore, 3MMD achieved significantly greater viral load suppression than 6MMD, and viral suppression was similar among those on 6MMD and 12MMD. Lastly, most of the studies were conducted in Africa.

Based on these findings, further studies outside Africa should be conducted to validate the current results, primarily to enable comparisons that yield generalizable findings to inform ART refill policies. Furthermore, our findings should be interpreted with caution, given the high variability among these studies, as evidenced by the heterogeneity score. Our findings have implications for ART refill, at least in Africa. Regulatory bodies, such as the WHO (< 1000 copies/mL) and HIV.org (< 200 copies/mL), have defined viral suppression differently, which may explain why countries lack a unified definition or guidelines on who should be placed on MMD. We encourage governing bodies to develop a unified definition of viral suppression, and we expect that Ministries of Health or equivalent parastatals globally will adhere to these guidelines. This would allow future studies to operationalize MMD and viral suppression uniformly and allow for adequate comparison of MMD’s effect on the HIV care continuum, including viral suppression, between and within settings, countries, and regions.

We also offer some programmatic recommendations based on our findings. The small marginal advantage of MMD over SoC indicates that MMD alone cannot drive a large improvement in virologic outcomes. Therefore, HIV clinics should implement MMD for stable PLWH as part of differentiated services delivery models and those at risk of treatment failure on SoC (i.e., monthly refill), while also ensuring adherence club, motivational adherence counselling, and early detection of PLWH at risk of treatment failure. HIV clinics that choose to implement MMD should provide no more than 3MMD (i.e., 3 bottles of antiretroviral therapy medication), as viral suppression is more likely than with 6MMD, and 6MMD and 12MMD have similar effects on viral loads. Lastly, HIV clinics that intend to implement MMD should look beyond the advantages it offers to them and their clients and consider how MMD can impact their supply chain strategy, particularly their storage capacity.

## Data Availability

Our study relies on published data. The supporting data for our findings are included in this article. All data obtained from original papers, along with related tables and figures, are available for sharing.
